# Use of butorphanol and diprenorphine to counter respiratory impairment in the immobilised white rhinoceros (*Ceratotherium simum*)

**DOI:** 10.4102/jsava.v89i0.1683

**Published:** 2018-10-18

**Authors:** Leith C.R. Meyer, Andrea Fuller, Markus Hofmeyr, Peter Buss, Michele Miller, Anna Haw

**Affiliations:** 1 Department of Paraclinical Sciences, University of Pretoria, South Africa; 2 School of Physiology, University of the Witwatersrand, South Africa; 3 Great Plains Conservation and Rhino without Borders, Maun, Botswana; 4 Veterinary Wildlife Services, South African National Parks, South Africa; 5 Department of Production Animal Studies, University of Pretoria, South Africa; 6 Division of Molecular Biology and Human Genetics, Stellenbosch University, South Africa

## Abstract

Opioid-induced immobilisation results in severe respiratory impairment in the white rhinoceros. It has therefore been attempted in the field to reverse this impairment with the use of opioid agonist-antagonists, such as nalorphine, nalbuphine, butorphanol and diprenorphine; however, the efficacy of some of these treatments has yet to be determined. The efficacy of butorphanol, either alone or in combination with diprenorphine both with and without oxygen insufflation, in alleviating opioid-induced respiratory impairment was evaluated. The study was performed in two parts: a boma trial and a field trial. Rhinoceroses were immobilised specifically for the study, according to a strict protocol to minimise confounding variables. A two-way analysis of variance was used to compare the physiological responses of the rhinoceroses to the different treatments and their effects over time. The intravenous administration of butorphanol (at 3.3 mg per mg etorphine) plus diprenorphine (at 0.4 mg per mg etorphine) did not offer any advantage over butorphanol (at 15 mg per mg etorphine) alone with regard to improving P_a_O_2_, P_a_CO_2_ and respiratory rates in etorphine-immobilised white rhinoceroses. Both butorphanol + diprenorphine + oxygen and butorphanol + oxygen, at the doses used, significantly improved the etorphine-induced hypoxaemia in both boma- and field-immobilised white rhinoceroses. Clinically acceptable oxygenation in field-immobilised white rhinoceroses can be achieved by using either treatment regimen, provided that it is combined with oxygen insufflation.

## Introduction

The ability to safely immobilise white rhinoceroses for management procedures such as translocation, veterinary treatments and fitting of tracking devices is essential for the long-term survival of these mega-herbivores, especially with increased poaching pressure. The advent of potent mu-opioid agonists, such as etorphine hydrochloride, significantly decreased the mortality rate of rhinoceroses during capture events (Alford, Burkhart & Johnson 1974). However, a drawback of the use of potent opioid agonists is that they impair respiration profoundly, especially in the white rhinoceros (Burroughs et al. 2012a).

Over the last 40 years, wildlife veterinarians have used opioid agonist-antagonists such as nalorphine, nalbuphine, butorphanol and diprenorphine in an attempt to reverse respiratory impairment when etorphine is used while maintaining adequate immobilisation (Boardman et al. 2014; Burroughs et al. 2012a; Bush et al. 2004; Fahlman 2008; Miller et al. 2013; Wenger et al. 2007). Before 2001, nalorphine was readily available in South Africa and the combination of nalorphine with diprenorphine for the partial antagonism of etorphine in white rhinoceroses was a protocol used in the Kruger National Park (Markus Hofmeyr, pers. comm., 23 Feb. 2012). However, the physiological benefits of administering this combination had never been scientifically evaluated, and even when nalorphine was used on its own as a partial antagonist in immobilised rhinoceroses, it only slightly improved blood oxygenation (Bush et al. 2004). Today, butorphanol and diprenorphine are the most widely accessible of the opioid agonist-antagonists, but conflicting opinions exist as to which drug, or combination of drugs, provides the best respiratory support for immobilised white rhinoceroses. As nalorphine is not available, some veterinarians now use a combination of diprenorphine and butorphanol. We hypothesised that butorphanol with diprenorphine would reduce respiratory impairment to the same extent as butorphanol (without diprenorphine) whether or not oxygen insufflation was administered.

## Research method and design

The project was divided into two parts: a boma study and a field study.

### Boma study: Study area and sample population

Eight sub-adult male white rhinoceroses were captured in the Kruger National Park (24.98984 S, 31.59263 E, alt. 1020 m) and housed individually in bomas. Husbandry procedures as per the South African National Parks (SANParks) standard operating procedures (SOPs) for boma-kept rhinoceroses were carried out throughout the experimental period. The trial started after a 1-month adaptation period and data collection occurred from August to December 2012. All immobilisations took place in the morning (between 05:20 and 10:50) and barometric pressure ranged from 737.0 mmHg to 745.9 mmHg.

### Chemical immobilisation and experimental interventions

At each trial, the rhinoceros was immobilised with a combination of 2 mg – 3 mg etorphine hydrochloride (dose range 2.0 *µ*g/kg – 2.6 *µ*g/kg) (M99^®^, Novartis, Kempton Park, South Africa, 9.8 mg/mL), 30 mg – 45 mg azaperone (dose range 30 *µ*g/kg – 40 *µ*g/kg) (Stressnil^®^, Janssen Pharmaceutical Ltd., Halfway House, South Africa, 40 mg/mL) and 2500 international units (i.u). hyaluronidase (lyophilised hyalase, Kyron Laboratories, Benrose, South Africa). The dosage of the drug was calculated from the previously measured body mass according to a standardised dose table (Haw et al. 2014). Immobilising drugs were administered into the nuchal hump using a 3 mL plastic dart with a 60 mm needle, powered by a CO_2_-powered dart-gun (Dan-Inject, South Africa) as described previously (Haw et al. 2014). Once the rhinoceros was immobilised, it was blindfolded and positioned in lateral recumbency (Time 0). Trials were performed only if the animal became recumbent within 15 min of darting, and position was altered between left and right lateral recumbency at each immobilisation.

The experiment consisted of five trials with the following interventions (Table 1): butorphanol IV (15 mg per mg etorphine, mean dose 33 *µ*g/kg, Kyron Laboratories, 20 mg/mL) (But), butorphanol IV (3.3 mg per mg etorphine, mean dose 7.5 *µ*g/kg) + diprenorphine IV (0.4 mg per mg etorphine, mean dose 0.89 *µ*g/kg, M5050^®^, Novartis, 12 mg/mL) (But+M5050), butorphanol IV (15 mg per mg etorphine) + oxygen intra-tracheal insufflation (AFROX, Johannesburg, South Africa, 30 L/min) (But+O_2_), butorphanol IV (3.3 mg per mg etorphine) + diprenorphine IV (0.4 mg per mg etorphine) + oxygen intra-tracheal insufflation (30 L/min) (But+M5050+O_2_) and sterile water (control) were evaluated and compared as measures to support the respiratory physiology of the immobilised white rhinoceros.

**TABLE 1:** Drugs and doses used to treat respiratory impairment in the rhinoceros.

Treatment intervention	Abbreviation
Butorphanol (15 mg/mg etorphine) (1.50 mL – 2.25 mL)	But
Butorphanol (3.3 mg/mg etorphine) + diprenorphine (0.4 mg/mg etorphine) (0.40 mL – 0.55 mL)	But+M5050
Butorphanol (15 mg/mg etorphine) + oxygen (30 L/min)	But+O_2_
Butorphanol (3.3 mg/mg etorphine) + diprenorphine (0.4 mg/mg etorphine) + oxygen (30 L/min)	But+M5050+O_2_
Sterile water (2 mL)	Control

Each rhinoceros received each intervention for the five trials in a randomised order at 2-week intervals. The interventions were administered 6 min after the rhinoceros became laterally recumbent. Clinical data and samples were collected at 5 min after the animal became laterally recumbent (before the intervention) and every 5 min thereafter for a 20-min immobilisation period. In the control trial, butorphanol (15 mg per mg etorphine) was administered at 21 min to facilitate arousal and loading into a crate.

### Clinical monitoring and data collection

Respiratory rate (breaths per minute) was monitored by counting thoracic and abdominal excursions and feeling for expired air at the nares. An immobilisation score, ranging from 1 (no immobilising or sedative effect) to 6 (excessive immobilisation depth with respiration < 3 breaths/min), was used to assess the level of immobilisation at 5, 10, 15 and 20 min. Level 3 indicated standing sedation that allowed handling, while levels 4 and 5 indicated recumbent immobilisation with or without ear movement, respectively.

Arterial blood samples for blood gas analyses were collected from the medial auricular artery catheterised with a 22G × 1″ IV catheter (Nipro Safelet Cath, Nipro Corporation, Bridgewater, NJ). A 0.5 mL sample was collected anaerobically into 1 mL heparinised syringes at 5, 10, 15 and 20 min after the rhinoceros became laterally recumbent. Partial pressures of carbon dioxide (P_a_CO_2_) and oxygen (P_a_O_2_) were measured immediately using a portable pre-calibrated blood gas analyser with pre-calibrated blood gas cassettes (Roche OPTI CCA Analyzer + OPTI cassette B, Kat Medical, Johannesburg, South Africa). The blood gas variables are reported at a body temperature of 37 °C. Body temperature was measured with a thermocouple thermometer (BAT-12, Physitemp Instruments Clifton, New Jersey, United States [US]) probe inserted 10 cm into the rectum.

In the trials in which oxygen was administered, the oxygen was delivered at a constant flow rate of 30 L/min via nasotracheal intubation using an equine stomach tube (9.5 mm od × 213 cm, Kyron Laboratories) as described by Bush et al. (2004).

Twenty-one minutes into the recumbent period, the rhinoceros was stimulated to stand and was guided into a crate for weighing using a scale suspended from a vehicle-mounted crane. Approximately 35 min after the animal became laterally recumbent, the effects of etorphine were reversed using naltrexone (50 mg/mL, Kyron Laboratories) administered intravenously into an auricular vein at 20 times the etorphine dose. The rhinoceros was then released from the crate into its boma.

All rhinoceroses used in these trials were monitored using a standardised boma scoring system on a daily basis for changes in appetite, defaecation and behaviour (Miller et al. 2016). In addition, haematological and biochemical analyses were performed at the time of each immobilisation to assess any changes in health status.

### Field: Study area and sample population

The study animals were 22 sub-adult male white rhinoceroses, which were immobilised in Kruger National Park in February 2013 during which time barometric pressures ranged from 717.1 mmHg to 748.3 mmHg. All animals appeared healthy based on body condition and physical examination. As in the boma study, all rhinoceroses were immobilised with a combination of etorphine hydrochloride, azaperone and hyaluronidase and drug doses were calculated on estimated body mass according to the same dose table as used in the boma study. Animals were located and darted using a helicopter. Immobilising drugs were delivered remotely using the same darting equipment as in the boma study. If animals were not recumbent within 15 min of darting, they were excluded from the trial and the effects of the immobilising agent reversed immediately. As butorphanol used alone without oxygen, and butorphanol plus diprenorphine without oxygen, did not completely reverse hypoxaemia in the boma part of the study, it was decided not to test drug effects without oxygen supplementation in the field. Therefore, 6 min into the immobilisation period, the following interventions were performed: 14 rhinoceroses received But+O_2_ and 8 rhinoceroses received But+M5050+O_2_.

Clinical monitoring was carried out as in the boma study, but the immobilisation period was extended to 25 min; thus, an extra arterial blood sample and a set of recordings were taken at that time. The arterial blood samples were analysed using a portable pre-calibrated blood gas analyser with recalibrated test cards (EPOC^®^ Portable analyser system + EPOC^®^ BGEM test cards, Kyron Laboratories, Johannesburg, South Africa).

Twenty-six minutes into the recumbent period, the rhinoceros was stimulated to stand and walk into a weighing crate and body mass was recorded. Approximately 40 min after the rhinoceros became laterally recumbent, the effects of etorphine were completely reversed using the pure opioid antagonist naltrexone, administered intravenously into an auricular vein at 20 mg per mg etorphine. The rhinoceros was then released from the weighing crate.

### Data analysis

We used GraphPad Prism version 6.00 for Mac OS X (GraphPad Software Inc., San Diego, California, US) for statistical analyses. All results are reported as mean ± SD, and *p* < 0.05 was considered statistically significant. For the boma study, we used a repeated measures two-way analysis of variance (ANOVA) followed by Tukey’s multiple comparisons for two separate analyses. In the first analysis, we looked for differences between responses to But, But+M5050 and sterile water (control) at 5, 10, 15 and 20 min (trials without oxygen), while in the second analysis we looked for differences between responses to But+O_2_, But+M5050+O_2_ and sterile water (control) at 5, 10, 15 and 20 min (trials with oxygen). For the field study, we used a two-way ANOVA followed by Sidak’s multiple comparisons test to test for differences between responses to But+O_2_ and But+M5050+O_2_ at 5, 10, 15, 20 and 25 min. A Tukey’s post-hoc test was used to test for differences in values between time points within each trial.

### Ethical consideration

This project was approved by the Animal Use and Care Committee (approval HAWA1042) of South African National Parks (SANParks) as well as the Animal Ethics Committees of the University of the Witwatersrand (approval no. 2012/23/04) and the University of Pretoria (approval no. V087/13).

## Results

All boma-housed rhinoceroses were in good health, indicated by normal demeanour and normal eating and defaecation habits throughout the study period. Field rhinoceroses were also in good health at the time of darting, determined by observing normal behaviour from the helicopter before darting and evaluating body condition score. Each animal’s health status was confirmed by subsequent haematology and biochemistry analysis of blood samples collected at the time of each immobilisation. Rectal temperatures for boma-immobilised rhinoceroses ranged from 36.7 °C to 39.0 °C and 37.6 °C to 38.5 °C for field-immobilised rhinoceroses. The average body mass of the boma rhinoceroses was 1220 kg ± 148 kg. In the field study, the average body mass for the But+O_2_ group (*n* = 14) was 1272 kg ± 227 kg, while that for the But+M5050+O_2_ group (*n* = 8) was 1336 kg ± 339 kg. Most field rhinoceroses were estimated to be in the correct body mass bracket according to the dose table (Haw et al. 2014), although four rhinoceroses in the But+M5050+O_2_ group were estimated to be in one bracket lower than their actual mass. Despite the lower than recommended dose of etorphine, these rhinoceroses were immobile within 15 min of darting. Etorphine doses ranged from 2.0 mg to 3.5 mg and azaperone 30.0 mg to 52.5 mg. Hyaluronidase was kept constant at 2500 i.u. for each rhinoceros. The level of immobilisation did not change between the trials as the median immobilisation score at all time points across all trials was 4 (recumbent with ear movements). No acute mortality occurred in the rhinoceroses that were studied.

### Boma study

**FIGURE 1:**
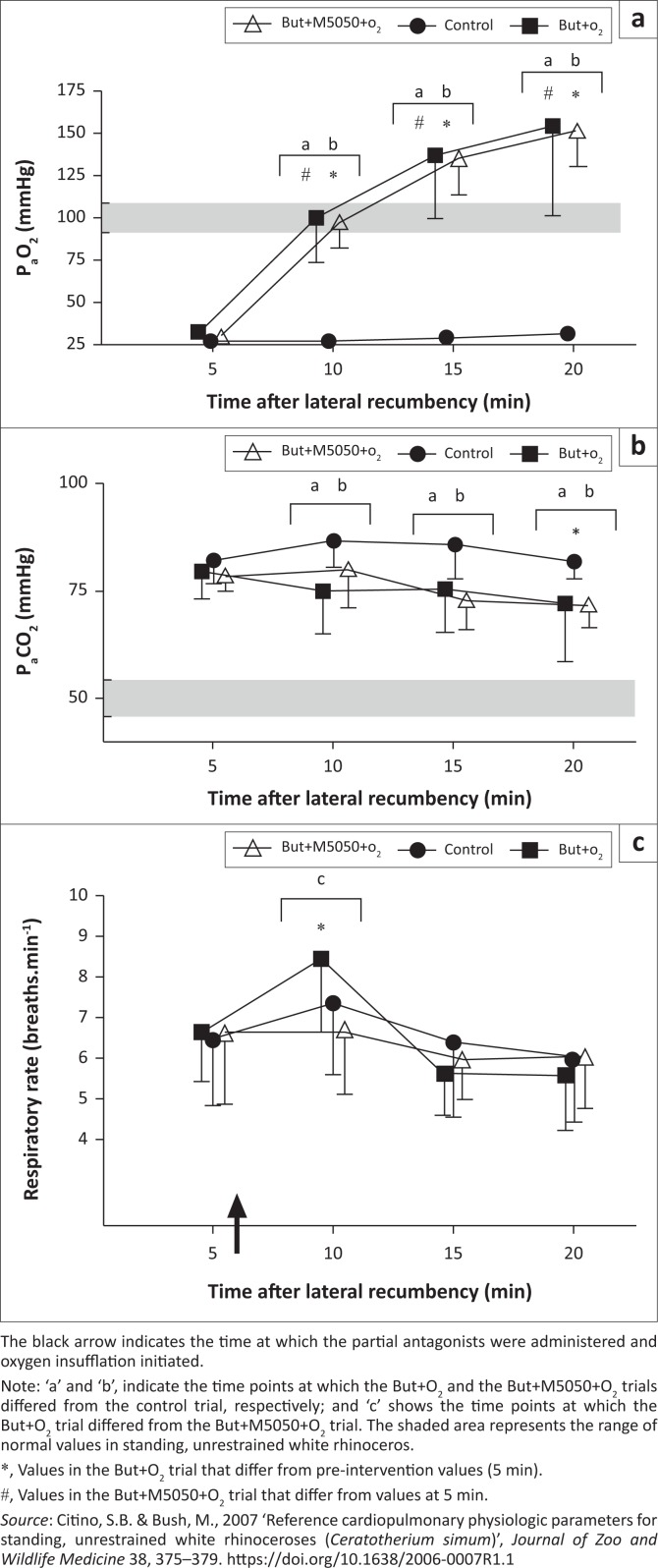
Boma study – Treatments with oxygen. (a) Partial pressure of arterial oxygen (P_a_O_2_), (b) partial pressure of arterial carbon dioxide (P_a_CO_2_) and (c) respiratory rate (breaths per minute) of boma-immobilised rhinoceros given intravenous butorphanol + oxygen insufflation (But+O_2_, *n* = 8), intravenous butorphanol + diprenorphine + oxygen insufflation (But+M5050+O_2_, *n* = 8) and sterile water (control, *n* = 8). Mean and standard deviations shown.

**FIGURE 2:**
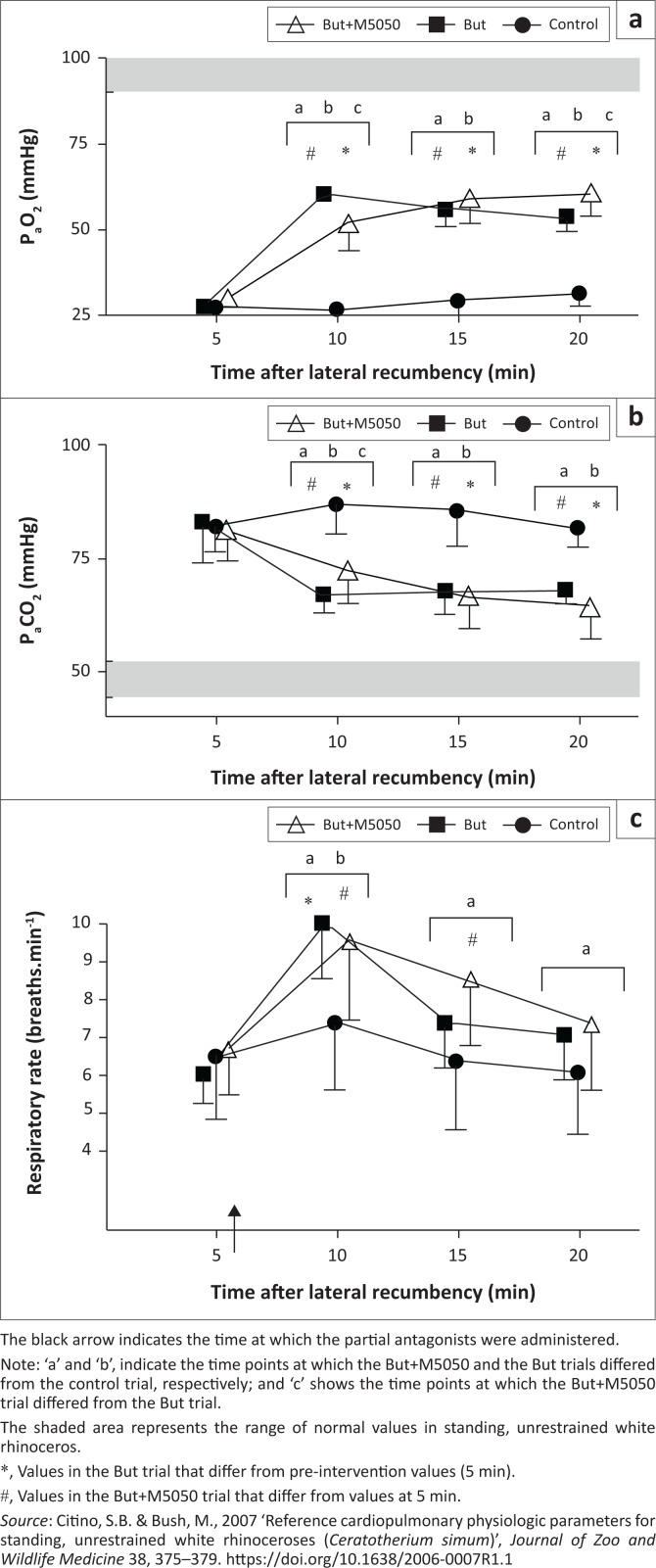
Boma study – Treatments without oxygen. (a) Partial pressure of arterial oxygen (P_a_O_2_), (b) partial pressure of arterial carbon dioxide (P_a_CO_2_) and (c) respiratory rate (breaths per minute) of boma-immobilised rhinoceros given intravenous butorphanol (But, *n* = 8), intravenous butorphanol + diprenorphine (But+M5050, *n* = 8) and sterile water (control, *n* = 8). Mean and standard deviations shown.

Chemical immobilisation resulted in severe hypoxaemia in all trials (Figure 1a and Figure 2a). At 5 min, the rhinoceroses had similar P_a_O_2_ values (*p* > 0.05) of 27 mmHg ± 7 mmHg (control), 28 mmHg ± 4 mmHg (But), 30 mmHg ± 6 mmHg (But+M5050), 31 mmHg ± 6 mmHg (But+O_2_) and 30 mmHg ± 7 mmHg (But+M5050+O_2_). P_a_O_2_ in the rhinoceroses then changed over time in both sets of trials, namely, the trials without oxygen (*F*_(3,21)_ = 216.3, *p* < 0.0001) and with oxygen (*F*_(3,21)_ = 81.64, *p* < 0.0001). At the time points following the treatment interventions, P_a_O_2_ differed amongst the trials without oxygen (*F*_(2,14)_ = 125.2, *p* < 0.0001) as well as those with oxygen supplementation (*F*_(2,14)_ = 49.52, *p* < 0.0001). Both But and But+M5050 led to improved oxygenation at 10, 15 and 20 min compared to the control trial, but the rhinoceroses were still hypoxaemic at 20 min (P_a_O_2_ = 54 mmHg ± 4 mmHg and 61 mmHg ± 6 mmHg in the But and But+M5050 trials, respectively). Similarly, both But+O_2_ and But+M5050+O_2_ treatments led to improved oxygenation at 10, 15 and 20 min compared to the control trial, but unlike the treatments without oxygen, these treatments completely corrected the etorphine-induced hypoxaemia. In these two trials with oxygen, the rhinoceroses had P_a_O_2_ values of 154 mmHg ± 53 mmHg (But+O_2_) and 152 mmHg ± 21 mmHg (But+M5050+O_2_) at 20 min. Throughout the immobilisation period, both the But+O_2_ and But+M5050+O_2_ treatment interventions led to similar P_a_O_2_ values in the rhinoceroses (*p* > 0.05). On the other hand, P_a_O_2_ values in the But and But+M5050 trials were statistically different at 10 min (60 mmHg ± 3 mmHg [But] and 52 mmHg ± 8 mmHg [But+M5050], *p* < 0.001) and 20 min (54 mmHg ± 4 mmHg [But] and 61 mmHg ± 6 mmHg [But+M5050], *p* < 0001), but not at 15 min (*p* > 0.05).

All rhinoceroses had similarly elevated P_a_CO_2_ values at 5 min of 82 mmHg ± 6 mmHg (control), 83 mmHg ± 9 mmHg (But), 81 mmHg ± 7 mmHg (But+M5050), 79 mmHg ± 7 mmHg (But+O_2_) and 72 mmHg ± 5 mmHg (But+M5050+O_2_) (Figure 1b and Figure 2b). P_a_CO_2_ then changed over time in the trials without oxygen (*F*_(3,21)_ = 20.8, *p* < 0.0001) and those with oxygen (*F*_(3,21)_ = 4.61, *p* = 0.01). P_a_CO_2_ in the rhinoceroses also changed amongst treatment groups in the two sets of trials (*F*_(2,14)_ = 34.16, *p* < 0.0001 [trials without oxygen]) and (*F*_(2,14)_ = 8.78, *p* = 0.003 [trials with oxygen]). The treatment interventions led to lower P_a_CO_2_ values in the rhinoceroses compared to when no treatment was administered (control) at all time points following the interventions, and by the end of the immobilisation period, P_a_CO_2_ values in the rhinoceroses that received treatments were lower compared to those at 5 min (*p* < 0.05). At 20 min, the rhinoceroses had P_a_CO_2_ values of 82 mmHg ± 4 mmHg (control), 68 mmHg ± 3 mmHg (But), 64 mmHg ± 7 mmHg (But+M5050), 72 mmHg ± 13 mmHg (But+O_2_) and 72 mmHg ± 5 mmHg (But+M5050+O_2_). Throughout the immobilisation period, there were no differences in the rhinoceroses’ P_a_CO_2_ values between the But and But+M5050 trials (apart from 10 min, *p* < 0.05), nor the But+O_2_ and But+M5050+O_2_ trials (*p* > 0.05).

At 5 min, the respiratory rates in the rhinoceroses were similar across trials (*p* > 0.05) with values of 7 ± 2 breaths/min (control), 6 ± 1 breaths/min (But), 7 ± 1 breaths/min (But+M5050), 7 ± 1 breaths/min (But+O_2_) and 7 ± 2 breaths/min (But+M5050+O_2_) (Figure 1c and Figure 2c)., which is much lower than the reported normal respiratory rates in standing, unsedated white rhinoceroses (16–23 breaths/min [Citino & Bush 2007]). The rhinoceroses’ respiratory rates then changed over time in both sets of trials (*F*_(3,21)_ = 14.76, *p* < 0.0001 [trials without oxygen] and *F*_(3,21)_ = 9.85, *p* = 0.0003 [trials with oxygen]). However, the different treatment interventions did not lead to differences in respiratory rates in the rhinoceroses (*F*_(2,14)_ = 3.4, *p* = 0.06 [trials without oxygen] and *F*_(2,14)_ = 0.1449, *p* = 0.87 [trials with oxygen]), except at 10 min, where the post-hoc test indicates that But+O_2_ led to improved respiratory rates compared to But+M5050+O_2_ (*p* < 0.05). At 20 min, the rhinoceroses had respiratory rates that were similar to those at 5 min across all trials, namely 6 ± 2 breaths/min (control), 7 ± 1 breaths/min (But), 7 ± 2 breaths/min (But+M5050), 6 ± 1 breaths/min (But+O_2_) and 6 ± 1 breaths/min (But+M5050+O_2_).

### Field study

**FIGURE 3:**
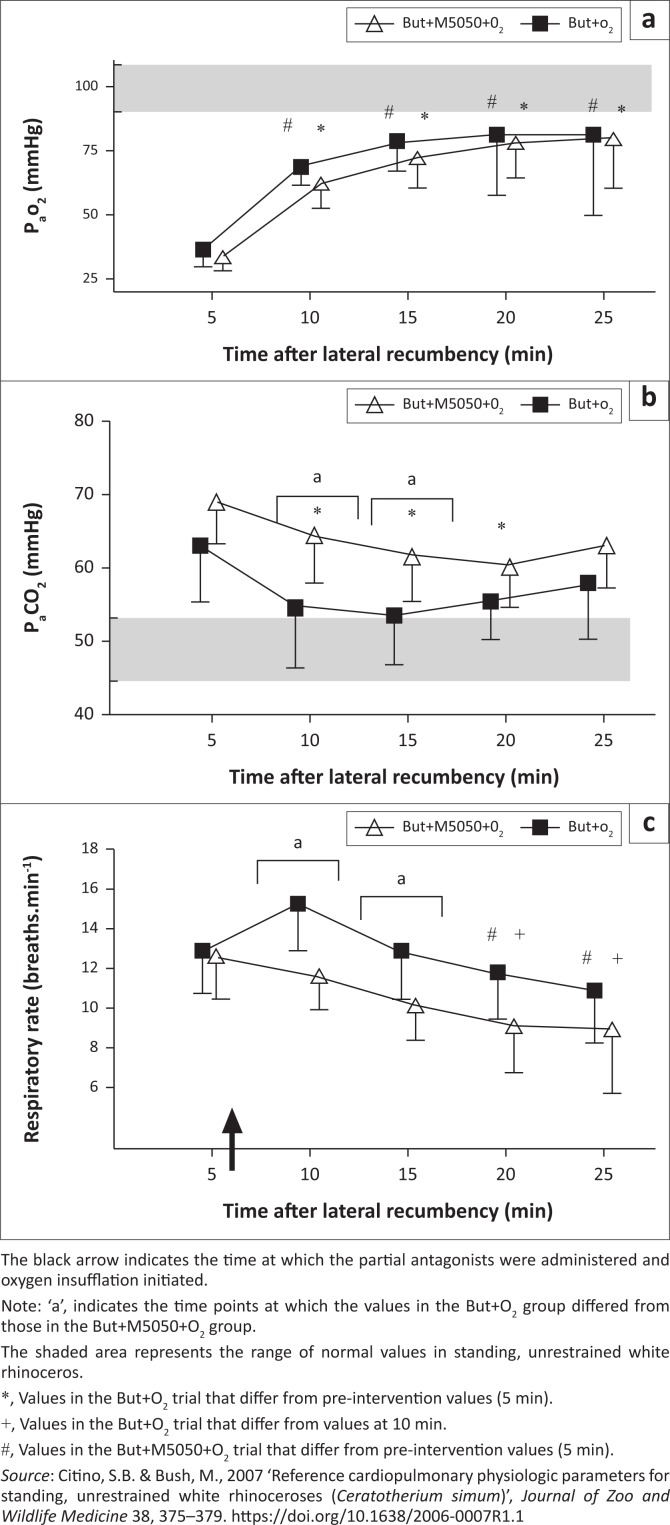
Field study. (a) Partial pressure of arterial oxygen (P_a_O_2_), (b) partial pressure of arterial carbon dioxide (P_a_CO_2_) and (c) respiratory rate of field-immobilised rhinoceros given intravenous butorphanol + oxygen insufflation (But+O_2_, *n* = 14) or intravenous butorphanol + diprenorphine + oxygen insufflation (But+M5050+O_2_, *n* = 8). Mean and standard deviations shown.

Similar to the boma study, chemical immobilisation with a combination of etorphine, azaperone and hyaluronidase led to severe hypoxaemia in all the rhinoceroses at 5 min post-recumbency (P_a_O_2_ = 35 mmHg ± 7 mmHg in the But+O_2_ trial and 34 mmHg ± 6 mmHg in the But+M5050+O_2_ trial) (Figure 3a). Both combinations of the partial-opioid antagonists combined with oxygen led to an immediate, positive effect on oxygenation. At 10 min, which is 4 min after the treatments, P_a_O_2_ was 69 mmHg ± 7 mmHg (*p* < 0.0001, 5 min vs 10 min) and 62 mmHg ± 10 mmHg (*p* = 0.009, 5 min vs. 10 min) in the But+O_2_ and But+M5050+O_2_ trials, respectively. Indeed, P_a_O_2_ was greater at all time points following the interventions, compared to that at 5 min, in both trials (*F*_(4,100)_ = 25.49, *p* < 0.0001) and there were no differences in the responses to the different treatments (*F*_(1,100)_ = 1.37, *p* = 0.244). Oxygenation in the rhinoceroses remained elevated throughout the immobilisation period with P_a_O_2_ values at 25 min of 81 mmHg ± 27 mmHg (But+O_2_) and 80 mmHg ± 20 mmHg (But+M5050+O_2_).

Together with the hypoxaemia, the rhinoceroses were also hypercapnic 5 min after they became recumbent, with P_a_CO_2_ values of 65 mmHg ± 8 mmHg (But+O_2_) and 69 mmHg ± 7 mmHg (But+M5050+O_2_) (Figure 3b). The P_a_CO_2_ in the rhinoceroses that received But+O_2_ then decreased (*F*_(1,100)_ = 26.04, *p* < 0.0001) to the extent that P_a_CO_2_ was lower (*p* < 0.05) than at 5 min at 10, 15 and 20 min, but not at 25 min. In the But+M5050+O_2_ trial, P_a_CO_2_ values were statistically similar at all time points (*p* > 0.05). At 10 min (*p* < 0.01) and 15 min (*p* < 0.05), the P_a_CO_2_ in the rhinoceroses that received But+O_2_ was lower than in those that received But+M5050+O_2_. At 10 min, P_a_CO_2_ values in the rhinoceroses were 58 mmHg ± 9 mmHg (But+O_2_) and 64 mmHg ± 7 mmHg (But+M5050+O_2_). By the end of the immobilisation period, P_a_CO_2_ values in the rhinoceroses were 60 mmHg ± 7 mmHg and 63 mmHg ± 6 mmHg in the But+O_2_ and But+M5050+O_2_ trials, respectively.

Corresponding with the observed hypercapnia, the respiratory rates were also different between treatments (*F*_(1,100)_ = 23.72, *p* < 0.0001) and across time (*F*_(4,100)_ = 7.98, *p* < 0.0001) (Figure 3c). Immobilisation caused hypopnea in all the rhinoceroses at 5 min (13 ± 2 breaths/min in both trials). But+O_2_ administration led to increased respiratory rates in the rhinoceroses compared to those that received But+M5050+O_2_ at 10 min (*p* < 0.01) and 15 min (*p* < 0.05). But+M5050+O_2_ administered to the rhinoceroses led to a decrease in respiratory rates at 20 min (9 ± 2 breaths/min, *p* < 0.05) and 25 min (9 ± 3 breaths/min, *p* < 0.05) compared to that at 5 min. On the other hand, But+O_2_ led to an initial clinically relevant but statistically insignificant increase in the respiratory rates of the rhinoceroses at 10 min (15 ± 2 breaths/min at 10 min vs. 13 ± 2 breaths/min at 5 min, *p* = 0.07), which was followed by a gradual decrease to the extent that the respiratory rates at 20 min (12 ± 2 breaths/min, *p* < 0.01) and 25 min (11 ± 3 breaths/min, *p* < 0.0001) were lower than the respiratory rates at 10 min.

## Discussion

Previous work has demonstrated that butorphanol with oxygen insufflation corrects the etorphine-induced hypoxaemia in boma-immobilised white rhinoceroses (Haw et al. 2014) and significantly reduces the severity of the hypoxaemia in field-immobilised white rhinoceroses to a clinically acceptable level (Haw et al. 2015). Here, we show that the same positive effect on blood gases can be achieved if butorphanol is replaced with a lower dose of butorphanol combined with diprenorphine. Similar to findings in our previous study (Haw et al. 2014), these opioid agonist-antagonists, at the doses used, only moderately improved blood oxygenation; thus, the addition of oxygen insufflation is necessary to correct the opioid-induced hypoxaemia. In the boma study described here, the partial pressure of arterial oxygenation was completely corrected with both treatments that included oxygen (But+O_2_ and But+M5050+O_2_), with no differences between the use of a single or combination of partial agonist-antagonist treatments. Although the interventions without oxygen (But and But+M5050) improved but did not correct the etorphine-induced hypoxaemia, the responses to the treatments were clinically similar in the boma trials. Similarly, in field-immobilised rhinoceroses, there were no differences in the rhinoceroses’ P_a_O_2_ values between the two treatments (But+O_2_ and But+M5050+O_2_), but the blood oxygenation was not completely corrected despite the animals receiving oxygen insufflation, although it was significantly improved, leading to a clear clinical benefit from the treatments.

The effects that the two partial antagonist treatments combined with oxygen had on the P_a_CO_2_ were not as profound as that on the P_a_O_2_. However, treatments led to a reduction in hypercapnia and there were no differences between the two treatments in the boma-immobilised rhinoceroses. This finding is consistent with previous work, which demonstrated that butorphanol with oxygen insufflation improved but did not fully correct etorphine-induced hypercapnia in boma-immobilised rhinoceroses (Haw et al. 2014). Also, consistent with previous findings (Haw et al. 2014), we demonstrated that the treatments without oxygen led to a more profound reduction in P_a_CO_2_, compared to the treatments with oxygen in boma-immobilised rhinoceroses. High levels of inspired oxygen have been found to increase P_a_CO_2_ levels in humans (Aboab et al. 2006; Perrin et al. 2011 Pilcher, Perrin & Beasley 2013), horses (Marntell, Nyman & Hedenstiernal 2005) and immobilised wildlife (Fahlman 2014) with respiratory compromise. A primary mechanism for the rise in P_a_CO_2_ with high inspired oxygen is likely worsening ventilation-perfusion mismatch caused by absorption atelectasis (Aboab et al. 2006; Marntell et al. 2005). Other proposed mechanisms could include a release of hypoxic vasoconstriction (increasing alveolar dead space), a decrease in the hypoxic ventilator drive (decreasing ventilation) or the Haldane effect (displacement of CO_2_ from haemoglobin into plasma) as a result of increasing PaO_2_ levels (Fahlman 2014). Perhaps, titrating the oxygen flow rate to achieve optimum P_a_O_2_ and P_a_CO_2_ levels, as recommended for humans with respiratory disorders (Pilcher et al., 2013), would result in lower P_a_CO_2_, while maintaining sufficient P_a_O_2_ in white rhinoceroses. In the field-immobilised rhinoceroses, this study shows that the But+O_2_ treatment led to a greater reduction in the hypercapnia than the But+M5050+O_2_ treatment. Concerning respiratory rate, the But+O_2_ treatment led to greater respiratory rates than the But+M5050+O_2_ treatment in both boma- and field-immobilised rhinoceroses. However, in the treatments without oxygen in boma-immobilised rhinoceroses, there were no differences in respiratory rate responses between the But and But+M5050 treatments at the doses used.

Both butorphanol and diprenorphine are opioid agonist-antagonists. However, diprenorphine is known to have greater antagonistic effects than butorphanol (Burroughs, Meltzer & Morkel 2012b). Indeed, diprenorphine (M5050^®^) is supplied with etorphine (M99^®^) by its current manufacturer, Voluplex, and is registered as the standard antidote for etorphine-induced immobilisation. To reverse the effects of etorphine, diprenorphine is given at 2–3 mg per mg etorphine (Burroughs et al. 2012b; Swan 1993). In most species, diprenorphine has been the most commonly used antagonist (Burroughs et al. 2012b) and recovery times following intravenous diprenorphine administration range from a few seconds to 4 min (Swan 1993). However, it is believed that diprenorphine does have some agonistic effects, and a degree of sedation may be maintained when it is used, particularly in some species (white rhinoceros, elephant and giraffe). Therefore, it is recommended that a pure opioid antagonist, such as naltrexone, be used to reverse the effects of opioid agonists in animals that are to be released into the wild (Burroughs et al. 2012b). In white rhinoceros, diprenorphine appears to not antagonise the effects of etorphine completely and animals will remain partially narcotised for up to 8 h following the administration of diprenorphine (Kock et al. 1995; Rogers 1993). Whether these effects can be attributed to possible agonistic effects of diprenorphine, as has been found at kappa-receptors in the guinea-pig ileum (Traynor, Corbett & Kosterlitz 1987), or simply poor efficacy or lower affinity of this drug to opioid receptors in this species remains to be determined.

Like butorphanol, diprenorphine has been advocated as a potential treatment to reverse the respiratory impairment effects of etorphine in immobilised white rhinoceroses. Therefore, the aim of this study was to compare the effects of butorphanol and butophanol plus diprenorphine because these treatments are currently used by veterinarians in the field to reduce the etorphine-induced respiratory impairment, but their efficacy in achieving this goal has not been rigorously evaluated or compared.

Based on anecdotal findings from the field, practitioners have suggested giving 1 mg diprenorphine with 10 mg butorphanol IV immediately to all white rhinoceroses (Burroughs et al. 2012a). To standardise our trial, diprenorphine and butorphanol were administered at a set ratio to the dose of etorphine used in the immobilising dart. Therefore, 0.4 mg diprenorphine and 3.3 mg butorphanol per mg etorphine were used, which equates to about 1 mg diprenorphine with 10 mg butorphanol for 2 mg – 3 mg etorphine administered.

The results of this study have demonstrated that the treatments But+O_2_ and But+M5050+O_2_ both elicited similar changes in respiratory responses and corrected the etorphine-induced hypoxaemia in boma-immobilised white rhinoceros. However, treatment with butorphanol+diprenorphine, at the doses used in these trials, was not as beneficial as butorphanol at the higher dosage used alone in correcting the etorphine-induced hypercapnia and hypopnoea. Our evidence shows that at certain times the rhinoceroses had higher P_a_CO_2_ values and lower respiratory rates after But+M5050+O_2_, compared to those after But+O_2_. Thus, although both treatments had the same effect on the rhinoceroses’ P_a_O_2_, the treatment with diprenorphine did not always improve P_a_CO_2_ as well as the treatment without diprenorphine, where a greater dose of butorphanol was used. This discrepancy in the treatments’ effects on P_a_CO_2_ may partly be explained by uncontrolled confounders. One possible limitation of the study is that the But+M5050+O_2_ group had a smaller sample size than that of the But+O_2_ group. Another possible explanation could be that when butorphanol+diprenorphine is used in the field, compared to butorphanol alone, the lower dose of butorphanol or the differing antagonist effects of diprenorphine in this combination may have a better effect on antagonising other pathophysiological effects of etorphine that cause hypoxaemia (Buss et al. 2018; Meyer et al. 2015), with less of an effect on ventilation or the production of carbon dioxide from metabolism.

## Conclusion

Regardless of the above-mentioned potential confounders, the results of this study have shown that using supplemental oxygen with a combination of low-dose butorphanol and diprenorphine offers no advantage, other than a potential cost-saving benefit, in alleviating etorphine-induced respiratory impairment compared to butorphanol (15 mg per mg etorphine) with oxygen. Some veterinarians are using diprenorphine on its own to reverse the etorphine-induced respiratory impairment in the white rhinoceros. Based on the results of these trials, it may be worthwhile to determine if a higher dose of diprenorphine used alone could offer any physiological benefit over butorphanol alone. However, this study confirms that arterial oxygen levels can be restored to clinically acceptable values in immobilised white rhinoceroses administered either But+O_2_ or But+M5050+O_2_ at the doses used in this study.
